# Surgery, Science, and Stewardship: The Changing Profile of the Abdominal Transplant Surgeon

**DOI:** 10.7759/cureus.97938

**Published:** 2025-11-27

**Authors:** Dimitrios Moris, Emmanouil Giorgakis

**Affiliations:** 1 Surgery, Duke University Medical Center, Durham, USA; 2 Surgery, University of North Carolina at Chapel Hill, Chapel Hill, USA

**Keywords:** abdominal transplantation, evolution, future directions, history, organ transplantation and challenges

## Abstract

Abdominal organ transplantation has undergone remarkable evolution over the past several decades, with clinical outcomes improving steadily and procedure volumes rising in parallel. Despite this progress, the abdominal transplant workforce is shrinking, and interest in fellowship training has noticeably waned, even as surgeons in practice report increasing levels of professional strain. The core challenge is that the responsibilities and expectations of the modern abdominal transplant surgeon are expanding far more rapidly than the field’s capacity to train, recruit, and retain its workforce. Emerging innovations, including advanced preservation techniques, ex vivo cellular and genomic interventions, sophisticated immune profiling, and more individualized immunosuppression strategies, are reshaping the discipline, although many of these technologies remain in early development or under preliminary regulatory evaluation. This editorial examines the trajectory of abdominal transplantation, the expanding demands placed on contemporary transplant surgeons, and the workforce, ethical, and scientific pressures that risk widening the gap between the field’s aspirations and its practical capacity. We conclude with recommendations aimed at ensuring that the future of abdominal transplantation remains both scientifically ambitious and professionally sustainable.

## Editorial

Introduction

The abdominal transplant surgeon embodies one of the most multifaceted professional identities in modern medicine. Abdominal organ transplantation, including kidney, liver, pancreas, intestinal, and multivisceral procedures, has progressed from experimental efforts conducted with limited supportive technologies in the mid-20th century to highly structured therapies with predictable outcomes supported by national registries [1]. The professional evolution of the transplant surgeon has been shaped by surgical innovation, immunological discovery, legislative frameworks, bioengineering, and team-based care models. Our central thesis is that the professional role of the abdominal transplant surgeon has expanded more rapidly than the systems designed to train, support, and sustain them, creating a widening structural mismatch that threatens long-term sustainability.

Why this viewpoint now

This reflection on the evolution of the abdominal transplant surgeon is especially timely. In recent years, surveys have demonstrated declining interest in transplantation careers among general surgery residents, with concerns centered on lifestyle demands, intensity of call schedules, and limited perceived work-life balance [2,3]. At the same time, transplantation has faced increased public scrutiny, fueled by media coverage questioning practices such as donation after circulatory death (DCD), debates about organ procurement organization performance, and broader mistrust in medical systems [4]. Against this backdrop, articulating the historical achievements, ongoing innovations, and enduring rewards of a career in abdominal transplantation is critical. The profession must not only adapt technologically and scientifically but also reaffirm its societal value, inspire the next generation of surgeons, and strengthen public trust in organ donation.

Origins of abdominal transplantation (1950s-1970s)

The foundations of modern transplantation were laid decades before the first clinical successes. Alexis Carrel’s Nobel Prize-winning work in vascular anastomosis and perfusion in 1912 established the technical underpinnings of organ transplantation [5]. Carrel’s later collaboration with Charles Lindbergh on early perfusion devices presaged modern machine perfusion. The first successful therapeutic organ transplant occurred in 1954 when Joseph Murray performed a kidney transplant between identical twins, demonstrating that organ transplantation could meaningfully restore physiological function [6]. Liver transplantation advanced through Thomas Starzl’s early efforts in the 1960s, although all initial grafts failed within weeks [7], prompting ethicists and clinicians to debate the procedure’s legitimacy. Pancreas transplantation, first performed by Kelly and Lillehei in 1966, initially experienced high mortality and graft loss but laid the foundation for the shift from bladder-drained to enteric-drained grafts in subsequent decades [8]. Early intestinal transplants in the 1960s and 1970s, primarily by Lillehei and colleagues, were largely unsuccessful due to rejection and sepsis [9,10]. During this era, surgeons bore full responsibility for perioperative management, immunosuppression, and critical care in settings lacking dialysis, advanced imaging, or dedicated ICUs, an intensity that shaped the profession’s identity.

Consolidation and professionalization (1980s-1990s)

The introduction of cyclosporine in the early 1980s marked a transformative shift, with kidney 1-year graft survival rising from roughly 50% to more than 80%, and liver patient survival improving from 30% to nearly 70% [11]. Tacrolimus in the 1990s produced further improvements in rejection rates and long-term graft function [12]. Parallel regulatory and organizational developments solidified transplantation as a distinct profession. The National Organ Transplant Act (NOTA) of 1984 established a national system of organ allocation [13], followed by the creation of the United Network for Organ Sharing (UNOS) in 1986 [14]. Professional consolidation occurred with the formation of the American Society of Transplant Surgeons (ASTS) in 1974, which established dedicated training pathways and professional standards [15]. Liver allocation reform accelerated with the introduction of the Model for End-Stage Liver Disease (MELD) score in 2002, which reduced waitlist mortality but generated controversy regarding hepatocellular carcinoma exceptions and geographic disparities [16]. These challenges prompted the 2020 implementation of acuity circles to reduce regional inequity.

Expansion, subspecialization, and globalization (2000s-2010s)

Living donor transplantation expanded dramatically during this period. Ratner’s landmark 1995 laparoscopic donor nephrectomy series demonstrated reduced donor morbidity and catalyzed widespread adoption of minimally invasive donor surgery [17]. However, donor mortality, estimated at 0.03% to 0.1% for kidney donors and higher for liver donors, remains a defining ethical responsibility [18]. The 2002 death of a living liver donor at Mount Sinai prompted major reforms in donor evaluation and institutional oversight [19]. Pancreas transplantation matured through the Minnesota group’s large series [20], while intestinal transplantation, despite its technical sophistication, continues to have the lowest long-term survival among solid organ grafts, with five-year graft survival below 50% in many registries [9,10,21]. Global expansion of transplantation was uneven. While activity increased in Asia, Latin America, and the Middle East, these developments were accompanied by concerns about organ tourism and commercialism. The Declaration of Istanbul (2008) established international ethical principles to combat organ trafficking [22], though enforcement varies substantially across regions.

The modern abdominal transplant surgeon (2020s-present)

The contemporary transplant surgeon operates within an increasingly complex ecosystem defined by rapid technological advances, rising patient acuity, and intensifying societal and institutional expectations.

Machine Perfusion and Ex Vivo Organ Modification

Machine perfusion has reshaped organ preservation and evaluation. Hypothermic and normothermic platforms, such as the LifePort Kidney system and the TransMedics Organ Care System, extend preservation times, reduce ischemia-reperfusion injury, and allow functional assessment prior to implantation [23]. Research centers are exploring therapeutic interventions during perfusion, including vector delivery, siRNA modulation, gene editing, and metabolic reconditioning of steatotic organs [24]. Anand’s recent Nature report demonstrated CRISPR-based xenogeneic kidney modification prior to transplantation into a brain-dead human recipient [25]. However, it is essential to emphasize that no FDA-approved gene-modified human solid organ transplantation exists, and all such procedures remain preclinical or conducted under Investigational New Drug (IND) regulation.

**Table 1:** Historical Evolution of Abdominal Organ Transplantation (1950s–2020s) A chronological framework illustrating how scientific, technical, ethical, and organizational developments shaped the identity and responsibilities of abdominal transplant surgeons. Future technologies, such as xenotransplantation, perfusion-based gene therapy, bioengineered grafts, remain experimental and may evolve unpredictably. The profession must prepare for multiple futures, including disruptive scenarios (e.g., manufactured organs reducing procedural volume).

Decade	Milestone/Development	Impact on Profession	Key Survival Metrics
Early 1900s	Carrel develops vascular anastomosis (1912 Nobel Prize)	Establishes technical feasibility; transplantation enters surgical imagination	—
1950s	First successful human kidney transplant (Murray, 1954; identical twins)	Demonstrates transplantation as therapeutic, not experimental	1-year kidney graft survival ~80% in identical twins
1960s	Starzl initiates orthotopic liver transplantation (1963); Kelly & Lillehei perform the first pancreas transplant (1966); early small-bowel attempts	High-risk, high-mortality surgery; surgeons assume total perioperative responsibility	1-year liver graft survival <30% in early series
1970s	Introduction of azathioprine and steroids; development of maintenance immunosuppression	Surgeons increasingly manage rejection pharmacology	Kidney 1-year graft survival rises to ~50%
1980s	Cyclosporine revolutionizes outcomes; NOTA (1984) and UNOS (1986) established	Professionalization; surgeons engage in national policy, ethics, and stewardship	Kidney 1-year graft survival >80%; liver >70%
1990s	Tacrolimus adoption; MELD score developed; expansion of pancreas transplantation	Surgeons become leaders in allocation, ethics, and hepatology	Liver 1-year graft survival >85%
2000s	Laparoscopic donor nephrectomy (Ratner et al.); living donor liver transplantation expands; 2002 donor death (Miller case) resets ethical oversight	Surgeons balance donor safety, minimally invasive technology, and non-therapeutic risk ethics	Living donor kidney mortality ~0.03%
2010s	Robotics enters donor surgery; intestinal/multivisceral programs mature but remain limited; global expansion	Hybrid identity: operator + systems innovator; global demand exceeds workforce	Intestinal 5-year graft survival <50%
2020s	Normothermic/hypothermic machine perfusion; early ex vivo gene-editing experiments; biomarker-guided immunosuppression; AI allocation pilots; xenotransplantation case reports (kidney 2024, heart 2022)	Surgeons evolve toward organ engineering, precision immunology, digital practice; workforce shortages intensify	NMP trials show ~20% reduction in early graft dysfunction

Precision Immunosuppression and Immune Monitoring

Immunosuppression is evolving toward individualized management. CYP3A5 genotyping predicts tacrolimus metabolism, with CYP3A5*1 expressers requiring 1.5-2 times higher initial dosing [26]. Donor-derived cell-free DNA (dd-cfDNA) has become integrated into routine monitoring at many centers, supported by the COPE and DART trials demonstrating early detection of alloimmune injury [27]. However, variability in test thresholds, limited insurance coverage, and the absence of trial-proven outcome superiority require cautious interpretation. Cellular immune assays such as ELISPOT and regulatory T-cell profiling (e.g., FOXP3 expression) remain primarily research tools [28]. The LIFT trial demonstrated that operational tolerance could be induced in approximately 20% of carefully selected liver recipients, though acute rejection rates ranged from 10% to 30% and required prompt reinstatement of immunosuppression [29].

**Table 2:** Machine Perfusion and Ex Vivo Organ Engineering Clinical and experimental applications of perfusion platforms and their implications for the evolving role of transplant surgeons. Gene-modified solid organs have not been transplanted into humans outside early experimental xenotransplantation contexts. FDA regulatory pathways remain undeveloped for gene-edited human allografts. Daytime scheduling benefits graft logistics but requires ethical scrutiny to avoid privileging clinician convenience over patient urgency.

Perfusion Method	Clinical/Experimental Applications	Surgeon’s Role	Key References
Hypothermic oxygenated perfusion (HOPE/DHOPE)	Reduces ischemia–reperfusion injury; protects biliary epithelium; improves outcomes in marginal liver grafts	Determines graft suitability; applies perfusion protocols; evaluates metabolic markers	Schlegel et al. [24]; van Rijn et al. [30]
Normothermic machine perfusion (NMP)	Viability testing; lactate clearance assessment; extended preservation; defatting of steatotic grafts; facilitates controlled, daytime implantation*	Real-time functional decision-making; graft optimization; procedural scheduling (with ethical oversight)	Nasralla et al. [23]
Ex vivo vector/gene therapy delivery	Experimental delivery of siRNA, AAV vectors, and CRISPR constructs to modulate inflammation, repair injury, or alter immunogenicity	Surgeon-scientist collaborates with immunology/engineering teams as an interventional organ biologist	DeMatteo et al. [31]; ongoing Phase I studies
Perfusion-assisted therapeutic interventions	Antibiotic perfusion; anti-inflammatory cocktails; mitochondrial protectants; ex vivo defatting	Bridges basic science, perfusion technology, and clinical care	Various experimental trials

Workforce Sustainability

The workforce crisis represents one of the field’s most urgent challenges. ASTS data demonstrate a 35-40% decline in fellowship applications since 2014 [3]. Burnout rates among transplant surgeons range between 56% and 60%, significantly higher than most surgical subspecialties [32]. Increasing use of marginal organs, administrative responsibilities, and the expectation of flawless outcomes with lower costs further strain the workforce.

Transplantation’s broader scientific and societal impact

Transplantation has saved hundreds of thousands of lives worldwide and remains among the most cost-effective therapies in medicine. Kidney transplantation offers substantial survival benefit and cost savings compared with dialysis [33]. Scientific contributions include Medawar's foundational immunology work on tolerance [34], Borel’s discovery of cyclosporine [35], and decades of ethical discourse on brain death, allocation fairness, and living donor autonomy. Despite advances, access remains constrained by persistent organ scarcity and inequity in allocation, emphasizing that the ultimate value of technological innovation will be determined by its ability to meaningfully expand and equitably distribute transplant opportunities.

Future directions and their uncertainty

The next decades of transplantation may be shaped by xenotransplantation, regenerative medicine, and artificial intelligence. Recent xenotransplantation cases-including the 2022 University of Maryland pig heart and the 2024 MGH and UAB pig kidney cases-demonstrated short-term function but were limited by complications such as porcine cytomegalovirus transmission and acute rejection [36]. No bioengineered solid organ has yet achieved durable human engraftment outside of early attempts involving tracheal and bladder constructs, and substantial scientific and regulatory barriers remain [37]. Artificial intelligence is beginning to inform allocation scoring, graft failure prediction, and perioperative decision support, though most tools remain investigational [38]. Robotics is expanding gradually, primarily in living donor nephrectomy and select kidney transplant implantation, with heterogeneity across platforms and limited long-term outcome data [39]. Given these uncertainties, workforce preparation must allow for multiple possible futures rather than assuming linear technological progression.

Conclusions

The abdominal transplant surgeon has evolved from a pioneering generalist into a multidisciplinary leader encompassing surgery, immunology, engineering, policy, ethics, and stewardship. Yet the field faces its greatest challenge: the rapidly expanding expectations for transplant surgeons far exceed the profession’s current capacity to train, sustain, and retain its workforce. Securing transplantation’s future requires coordinated reform. Training pathways must evolve toward competency-based, modular structures that incorporate digital literacy, biological understanding, and ethical reasoning. Workforce sustainability requires national monitoring of staffing needs, restructuring of call burdens, and institutional recognition of clinical excellence as a legitimate pathway for promotion. Ethical stewardship must include expansion of organ donation, reduction of allocation disparities, and vigilance against organ trafficking. Global collaboration must support brain-death legislation, infrastructure development, and training partnerships in low- and middle-income countries. The specialty’s longevity will depend not only on scientific innovation but on rebuilding a resilient workforce capable of advancing equitable, ethical, and effective transplantation for the next generation.
